# Ethanol infusion of the vein of Marshall reduces localized voltage but preserves mitral isthmus conduction velocity

**DOI:** 10.3389/fcvm.2026.1807237

**Published:** 2026-04-21

**Authors:** Yi Lu, Li Shu, Chunhui Liu, Shenghui Ma, Zhejun Cai

**Affiliations:** 1 Department of Cardiology, The Second Affiliated Hospital, School of Medicine, Zhejiang University, Hangzhou, China; 2 State Key Laboratory of Transvascular Implantation Devices, Hangzhou, China; 3 Heart Regeneration and Repair Key Laboratory of Zhejiang Province, Hangzhou, China

**Keywords:** atrial fibrillation, ethanol infusion, mitral isthmus ablation, perimitral conduction velocity, vein of Marshall

## Abstract

**Background:**

Ethanol infusion of the vein of Marshall (EIVOM) has emerged as a promising adjunct to pulmonary vein isolation (PVI) for atrial fibrillation (AF).

**Methods:**

Patients undergoing catheter ablation for persistent AF (>3 months) were enrolled in this study. After PVI, high-density mapping (HD-Grid, Abbott, St Paul, MN) was performed during coronary sinus pacing (100 bpm) to assess left atrial conduction velocity (CV) and voltage. EIVOM was then performed (1–3 mL ethanol × 3 doses), followed by repeat mapping. The CV and voltage were analyzed in the left lateral wall (mitral isthmus/appendage base), with ≥300 points collected per region. A post-EIVOM electrophysiological study with isoprenaline was conducted to induce arrhythmias.

**Results:**

Twenty patients with AF (mean age, 64.30 [SD, 7.97] years, five women [25.0%]) with 24 (interquartile range: 6–60) months of AF duration underwent EIVOM (7.8 ± 2.1 mL). Voltage dropped from 1.92 (1.12–2.61) to 1.24 (0.91–2.05) mV postprocedure (*p* < 0.05), particularly in the atrial ridge regions. CV remained unchanged (1.00 [0.92–1.10] vs. 1.02 [0.95–1.14] m/s, *p* = 0.15). Atrial flutter was induced in three patients (one mitral isthmus-dependent, one tricuspid isthmus-dependent, and one roof-dependent). All patients maintained sinus rhythm at discharge without complications.

**Conclusions:**

In the acute setting, EIVOM induces localized voltage reduction in the atrial ridge regions without markedly affecting conduction velocity in the mitral isthmus areas.

## Introduction

Persistent atrial fibrillation (AF) remains a challenge for initiating treatment through catheter ablation, with suboptimal long-term success rates being reported despite pulmonary vein isolation (PVI). The vein of Marshall (VOM), an embryological remnant of the left superior vena cava, has been implicated in the pathogenesis of AM. The VOM is located proximal to the valve of Vieussens and is directed posterolaterally toward the left atrium and pulmonary veins (1, 2). Previous studies have shown that the VOM serves as a potential source of AF triggers and acts as a tract for both parasympathetic and sympathetic innervations, contributing to the maintenance of AF (2, 3). However, catheter ablation of the VOM is rendered technically difficult because of its epicardial location and insulation by adipose tissue.

Ethanol infusion of the vein of Marshall (EIVOM), pioneered by Valderrábano et al., enhances ablation efficacy by targeting localized substrates and facilitating mitral isthmus block. Recent studies that included randomized controlled trials such as VENUS (4) and PROMPT-AF (5) have demonstrated that EIVOM, in addition to PVI, can increase the likelihood of a person remaining free of AF or atrial tachycardia. The growing interest in EIVOM calls for a better understanding of the effect of ethanol on the perimitral area. Although prior studies demonstrated voltage reduction in the endocardial VOM region post-EIVOM (6), its impact on perimitral conduction velocity (CV) remains unclear. Notably, in contrast to prior strategies that combined EIVOM with systematic mitral isthmus ablation, the present study evaluates the electrophysiological effects of EIVOM without routine mitral isthmus ablation. With the recently developed omnipolar technology (OT), we were able to analyze the voltage, activation direction, and CV independent of the catheter position (7). This study utilizes OT to evaluate the acute effects of EIVOM on atrial voltage and CV.

## Methods

### Study population

This prospective study enrolled 20 adult patients with non-valvular persistent AF (AF duration >3 months) who underwent catheter ablation at the Second Affiliated Hospital, Zhejiang University School of Medicine (December 2024 to March 2025). The exclusion criteria were as follows: (1) a history of cardiac ablation or cardiac surgery; (2) moderate-to-severe mitral stenosis or mitral insufficiency; (3) ischemic heart disease, congenital heart disease, and cardiomyopathy (such as amyloidosis, hypertrophic, and cardiomyopathy); (4) inability to maintain sinus rhythm after PVI and cardioversion during the procedure; and (5) left atrial (LA) appendage thrombus formation or other contraindications to catheter ablation or anticoagulation.

This study was approved by the Institutional Ethics Committee of the Second Affiliated Hospital, Zhejiang University School of Medicine.

### Procedural protocol

**Figure 1:**
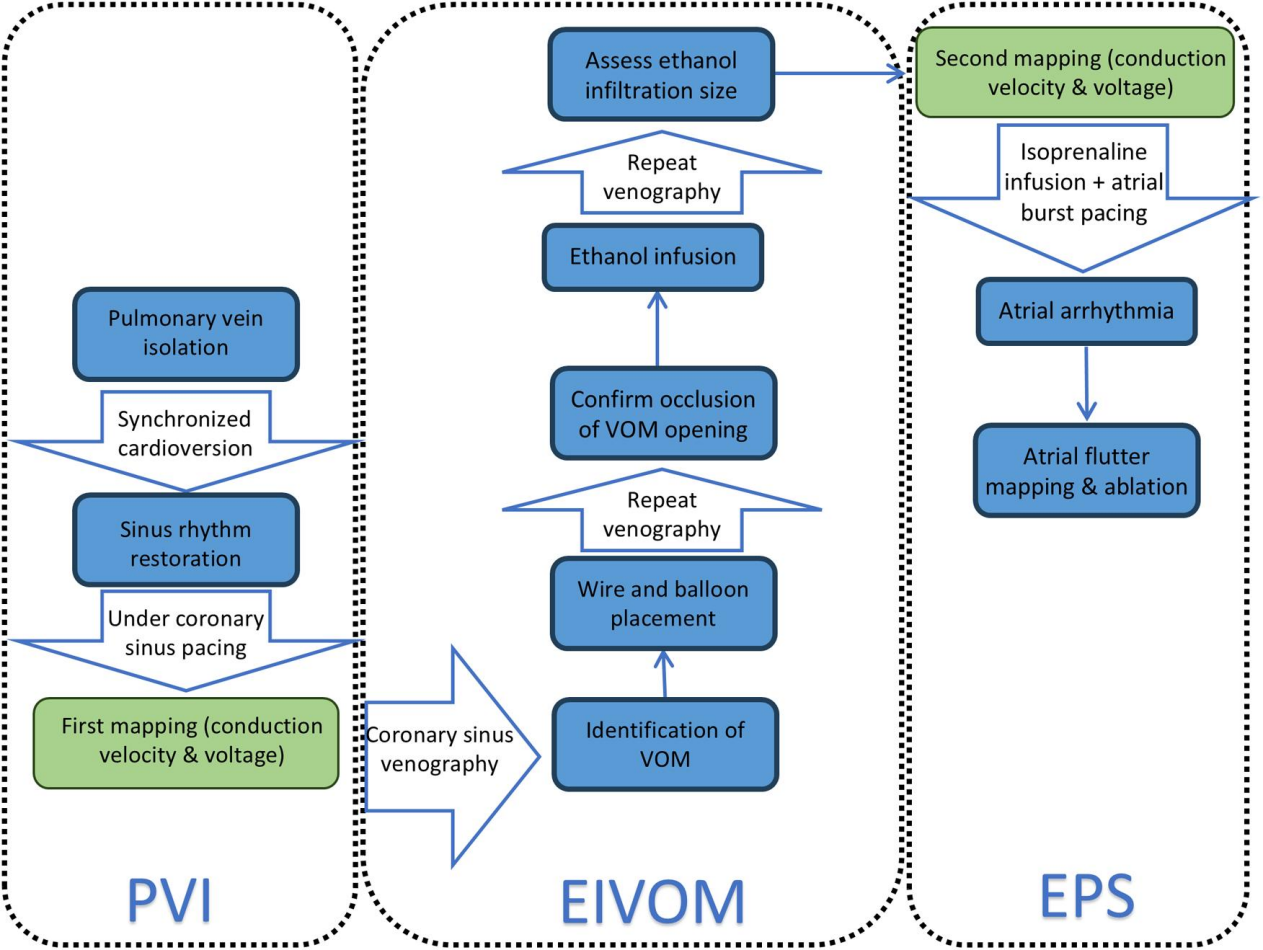
A procedural workflow of the integration of catheter ablation and ethanol infusion. PVI, pulmonary vein isolation; EIVOM, ethanol infusion of the vein of Marshall; EPS, electrophysiological study.

The procedural workflow integrating catheter ablation and ethanol infusion is shown in Figure 1. All patients underwent PVI using an irrigated ablation catheter (ThermoCool SmartTouch SF Catheter, Abbott, St Paul, MN) guided by the EnSite X system. Sinus rhythm was restored via synchronized cardioversion (≤3 attempts) after PVI. A three-dimensional conduction velocity and voltage mapping of the LA was performed under coronary sinus pacing (usually from CS 5 to 6, with 100 bpm) using a high-density mapping catheter (HD-Grid, Abbott, St Paul, MN), with the following settings: filter setting = 30–300 Hz, cycle length stability = 20 ms, catheter position stability = 1 mm, point density = 1 mm, internal projection = 10 mm, external projection = 10 mm, and interpolation = 10 mm.

EIVOM was performed subsequently as described previously (6). Coronary sinus venography was performed to identify the presence of the VOM. An angioplasty wire (Whisper 0.014, Abbott or Sion Blue 0.014, Asahi) was inserted into the VOM as far as possible to guide the advancement of the over-the-wire balloon (MINI TREK, 1.5–2 mm diameter and 6–15 mm length, Abbott) within its proximal portion. Venography was repeated after the inflation of the balloon and retrievement of the guiding wire to confirm the occlusion of the opening of the VOM. Absolute ethanol was administered three times, with each dose ranging between 1 and 3 mL (based on the size and depth of the VOM). Venography was required to be performed to demonstrate the infiltration size of ethanol before repeated conduction velocity and voltage mapping were conducted.

A thorough electrophysiological study with isoprenaline infusion and atrial burst pacing was conducted at the end of the second mapping with the aim to induce atrial arrhythmia, especially mitral isthmus-dependent atrial flutter. Atrial flutter was mapped using HD-Grid, and the critical isthmus was ablated.

### Measurements of electrical conduction velocity and voltage

The HD-Grid was used in each procedure to map and obtain details of conduction and voltage in the LA, especially in the left lateral wall (including the base of the LA appendage and the mitral isthmus region), as described previously (7, 8). The upper part contained the left atrial appendage and the warfarin ridge at its upper edge, while the lower part comprised the left inferior pulmonary vein ostium at its lower edge. The method for calculating the CV has been reported in detail in a previous study (9). A minimum of 300 points of CV and voltage data were collected in each part of the perimitral area, both pre- and post-EIVOM.

### Data collection

Baseline data were collected from the medical history of the patients, including their age, gender, past medical history, duration of atrial fibrillation, and BMI. Transthoracic echocardiography was performed before the procedure to rule out congenital heart diseases, cardiomyopathies, and valvular lesions and measure the anteroposterior diameter of the left atrium and the ejection fraction. Serum creatinine, potassium, and B-type natriuretic peptide (BNP) levels were collected on the day of the procedure.

### Statistical analyses

Data were presented as mean ± standard deviation (SD) or median (25th and 75th percentiles) for continuous variables, as appropriate. Data were expressed as the number (percentage) for qualitative variables. The *χ*^2^-test was used to compare categorical variables, and Student’s t-test or the Mann–Whitney U test was used for continuous variables between the two groups for normal and non-normal distribution data. All statistical analyses were performed using IBM SPSS Statistics for Windows, version 22.0 (IBM, Armonk, New York).

## Results

This study enrolled 20 participants, with a mean age of 64.30 ± 7.97 years. Women accounted for 25% (5/20) of the total study population. The median duration of AF was 24 months (interquartile range: 6–60 months). Hypertension was highly prevalent, affecting 65% (13/20) of the participants, while coronary artery disease was present in 10% (2/20) of the cohort. The median CHA2DS2-VASc score was 2, indicating a moderate-to-high risk of stroke in this AF population.

In terms of cardiac function and structure, the mean left ventricular ejection fraction was 60.5 ± 11.8%, while the mean LA size was 4.47 ± 0.38 mm. The median BNP level was 166 pg/mL (71.2–315.6 pg/mL), indicating a high prevalence of HFpEF among patients with AF.

**Figure 2:**
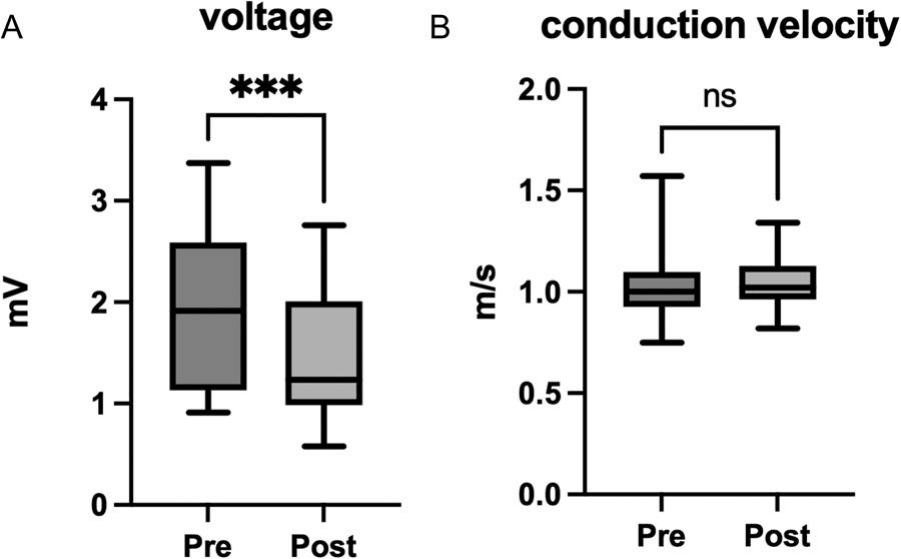
A comparison of voltage and conduction velocity in the left atrial lateral wall before and after EIVOM. **(A)** A voltage map shows a significant reduction in the lateral wall after EIVOM. **(B)** The conduction velocity in the lateral wall remains unchanged before and after EIVOM.

An average of 7.8 ± 2.1 mL of ethanol was injected into the vein of Marshall, resulting in 100% staining in this area. Detailed left atrial mapping was performed to evaluate the changes in the local activation time, CV, and voltage. We collected 471 (424, 567.5) and 435.5 (383.8, 563.8) data points in the left lateral wall area before and after EIVOM, respectively. Pre- and postprocedure mapping points in all patients are given in Table 1. The voltage dropped significantly after EIVOM from 1.92 (1.12–2.61) to 1.24 (0.91–2.05) mV (*p* < 0.05; Figure 2A). The distribution of low-voltage areas after EIVOM varied among different patients. The most affected regions were the atrial ridge, followed by the mitral isthmus, which could be explained by the course of the vein of Marshall.

**Table 1:** A summary of left atrial mapping points before and after the procedure in 20 patients.

Patient ID	Preprocedure mapping points	Postprocedure mapping points
Patient 01	5,807	5,877
Patient 02	18,108	18,612
Patient 03	20,712	10,402
Patient 04	10,284	9,504
Patient 05	16,729	10,403
Patient 06	25,452	23,292
Patient 07	20,072	14,904
Patient 08	12,744	7,991
Patient 09	16,560	12,168
Patient 10	13,604	13,356
Patient 11	12,682	11,855
Patient 12	6,480	13,500
Patient 13	13,955	13,608
Patient 14	18,984	14,508
Patient 15	17,520	20,726
Patient 16	14,956	14,256
Patient 17	9,792	16,942
Patient 18	17,686	19,978
Patient 19	4,536	7,272
Patient 20	12,298	12,636

This table includes all mapping points collected from the left atrium of 20 patients undergoing ablation. Preprocedure mapping points refer to those acquired before ablation, while postprocedure mapping points were collected after the procedure.

**Figure 3:**
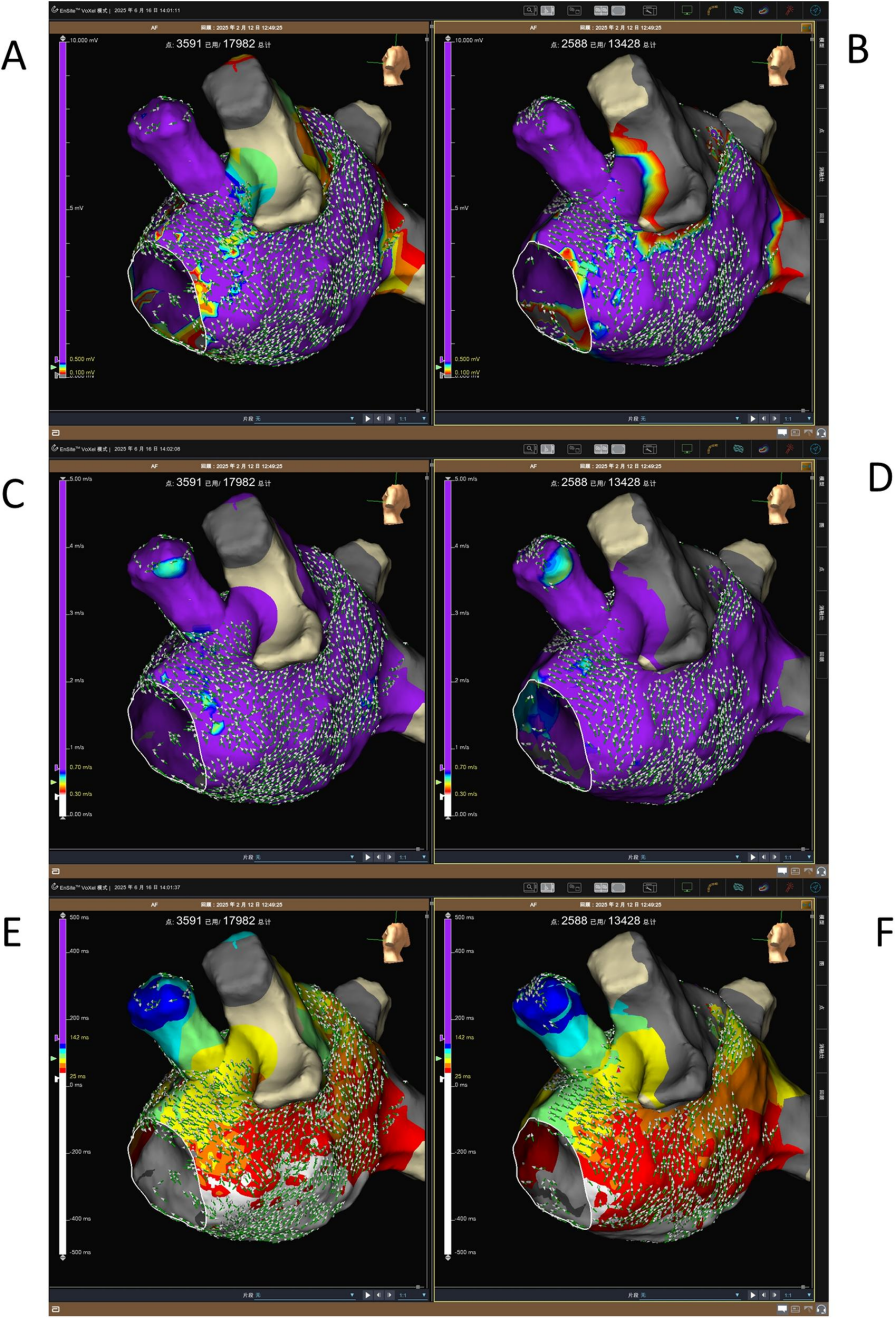
Voltage, conduction velocity, and activation maps from the same patient before and after EIVOM. **(A)** A pre-EIVOM voltage map showing the baseline left atrial voltage distribution. **(B)** A post-EIVOM voltage map showing a reduction in voltage, particularly in the atrial ridge of the lateral wall. **(C)** A pre-EIVOM conduction velocity map demonstrating baseline conduction characteristics. **(D)** A post-EIVOM conduction velocity map showing no significant change in the conduction velocity. **(E)** A pre-EIVOM activation sequence map illustrating the propagation pattern during baseline pacing. **(F)** A post-EIVOM activation sequence map indicating a similar activation pattern after EIVOM.

We then evaluated the conduction velocity in the same region by pacing from the middle of the coronary sinus. Interestingly, there was no significant change in the electric conduction velocity (*p* = 0.15). The mean CV was 1.00 m/s (0.92, 1.10) at baseline and 1.02 m/s (0.95, 1.14) after EIVOM (Figure 2B). Previous studies have reported that the cut-off value for abnormal CV in the atrium is between 0.61 and 0.90 m/s (7). In this study, we set the cut-off value for CV at 0.7 m/s. Figure 3 shows an example of the voltage map, activation map, and conduction velocity before and after EIVOM.

A thorough electrophysiological study with isoprenaline infusion and atrial burst pacing was conducted after mapping. Atrial flutter was induced in three patients, including one case of mitral isthmus-dependent, one case of typical tricuspid isthmus-dependent, and one case of roof-dependent atrial flutter. Notably, in the case of mitral isthmus atrial flutter, a clearly distinguishable scar was found in the anterior wall of the LA rather than the mitral isthmus, and the anterior wall was involved in the reentry circuit. No complications occurred during the procedure. All patients remained in sinus rhythm until discharge.

## Discussion

This is the first study to use OT mapping to characterize the acute effect of EIVOM on conduction in the per-mitral area. The results showed that EIVOM produced significant scarring on the warfarin ridge and mitral isthmus but did n't affect conduction in this region.

Ethanol can diffuse through cell membranes and cause cellular protein denaturation, resulting in immediate cell destruction. In a histological study of the ventricular myocardium, it was found that acute alcohol lesions generated myocyte dissolution, cellular vacuolization, and fiber disarray (10). Thus, EIVOM-related injury is assumed to be transmural and locates according to the vein trajectory and collateral network. A recent study showed that EIVOM could cause an immediate low-voltage area and permanent scarring at 3 months of follow-up. Conversely, in the same study, evaluation using cardiac MRI revealed that EIVOM-induced transmural “cell death” of cardiomyocytes did not affect atrial systolic function (10).

Because the left lateral wall is activated by the collision of the anterior and posterior wavefronts and by fibers with a more heterogeneous distribution under sinus rhythm (11), we used pacing from the adjacent coronary sinus to avoid these confounding factors. As confirmed in previous studies, our study demonstrated that the distribution of low-voltage areas was centered on the atrial ridge, followed by the mitral isthmus. This could be explained by the projection of the Marshall vein, as well as the fact that the occlusion of the OTW balloon in the Marshall vein was usually in the middle to distal region, rather than at the opening of the vein, which hampers the optimal ablation effect in the region close to the mitral valve. These findings might partly explain the finding in our study that EIVOM did n' affect the conduction speed in the perimitral valve region. Further studies are needed to precisely quantify the ethanol infiltration area and compare conduction velocity within the same region, in order to validate this hypothesis.

Another interesting finding of our study was that perimitral atrial flutter occurred only in a single patient after the combination of PVI and EIVOM. Because slow-conduction zones are usually involved in macro-re-entry atrial flutters (12), the absence of a slow-conduction zone after EIVOM is one of the reasons for the occurrence of perimitral atrial flutter in a single patient. In another study, investigators found that in patients with perimitral atrial flutter, there was often a discrepancy between the anatomic isthmus (low-voltage and slow-conduction zone) and the arrhythmogenic channels, and the septal-anterior wall was most commonly involved (13).

The events that really occur after EIVOM are yet to be clarified. In addition to benefits such as autonomic modulation and elimination of arrhythmogenic structures with the projection of the Marshall ligament (2, 3, 14), EIVOM facilitates the isolation of the left pulmonary veins, especially in areas like the warfarin ridge, where it is difficult for the ablation catheter to maintain good contact. Moreover, the VOM may provide epicardial connections between the pulmonary veins and the LA that are not amenable to conventional endocardial antral ablation (15). Studies that included RCTs such as VENUS (4) and PROMPT-AF (5) have demonstrated that mitral-isthmus block with the assistance of EIVOM could increase the likelihood of sinus rhythm maintenance beyond PVI; however, it could be argued whether mitral-isthmus block or simply EIVOM is responsible for these benefits. In a VENUS substudy, the favorable impact of EIVOM was potentiated when a perimitral block was achieved (16). Our study differs from these strategies of prior studies in the sense that systematic mitral isthmus ablation was not performed, and achieving mitral isthmus block was not an intended procedural endpoint. Therefore, our findings mainly reflect the acute electrophysiological effects of EIVOM. Whether a strategy of PVI combined with EIVOM alone without an intentional mitral line block can improve long-term outcomes compared with PVI alone remains to be determined. The benefit of PVI + EIVOM alone without a mitral line block has yet to be shown to improve outcomes over PVI and needs to be further demonstrated. In addition, perimitral atrial flutter subsequent to unblocked linear ablation lesions is the most common type of recurrent tachycardia (5, 17), and there was a high rate of non-blocked cases in the RCTs mentioned previously.

## Limitations

This study had several limitations. First, it was a single-center study that included a relatively limited number of subjects. Second, the absence of long-term follow-up may have led to an underestimation of the actual incidence of atrial arrhythmias, including perimitral atrial flutter, especially in patients who received EIVOM without additional mitral isthmus ablation. Third, conduction velocity is only an index reflecting the conduction property of the specific region, and the cut-off value of 0.9 m/s is not supported by large-volume data. Fourth, it has been demonstrated that the lesion area caused by EIVOM will expand over time (18); however, conduction velocity in our study was assessed only at a single time point, immediately after the procedure. Its time-dependent changes remain unclear and warrant longitudinal investigation. Fifth, owing to the invasive nature and complexity of epicardial access in this patient cohort, conduction velocity was assessed only on the endocardial surface, and epicardial conduction was not evaluated. Additional parameters such as the extent of energy-based ablation, detailed CS/VOM electrogram characteristics, and anatomical factors like mitral isthmus thickness may further contribute to the understanding of electrophysiological effects, and future studies incorporating the above aspects may provide additional mechanistic insights.

## Conclusion

In the acute setting, EIVOM effectively creates localized voltage reduction in the perimitral regions but does not significantly alter conduction velocity. These findings suggest that the therapeutic benefits of EIVOM in AF ablation may arise from substrate modification rather than perimitral conduction slowing.

## Data Availability

The raw data supporting the conclusions of this article will be made available by the authors without undue reservation.
